# T-Cell Therapeutics Targeting Human Parainfluenza Virus 3 Are Broadly Epitope Specific and Are Cross Reactive With Human Parainfluenza Virus 1

**DOI:** 10.3389/fimmu.2020.575977

**Published:** 2020-10-05

**Authors:** Katherine M. Harris, Sarah E. Horn, Melanie L. Grant, Haili Lang, Gelina Sani, Mariah A. Jensen-Wachspress, Vaishnavi V. Kankate, Anushree Datar, Christopher A. Lazarski, Catherine M. Bollard, Michael D. Keller

**Affiliations:** ^1^Center for Cancer and Immunology Research, Children’s National Hospital, Washington, DC, United States; ^2^Center for Cancer and Blood Disorders, Children’s National Hospital, Washington, DC, United States; ^3^GW Cancer Center, George Washington University, Washington, DC, United States; ^4^Division of Allergy and Immunology, Children’s National Hospital, Washington, DC, United States

**Keywords:** parainfluenza, immunotherapy, T-cell, bone marrow transplant, anti-viral

## Abstract

Human Parainfluenza Virus-3 (HPIV3) causes severe respiratory illness in immunocompromised patients and lacks approved anti-viral therapies. A phase I study of adoptively transferred virus-specific T-cells (VSTs) targeting HPIV3 following bone marrow transplantation is underway (NCT03180216). We sought to identify immunodominant epitopes within HPIV3 Matrix protein and their cross-reactivity against related viral proteins. VSTs were generated from peripheral blood of healthy donors by ex-vivo expansion after stimulation with a 15-mer peptide library encompassing HPIV3 matrix protein. Epitope mapping was performed using IFN-γ ELIspot with combinatorial peptide pools. Flow cytometry was used to characterize products with intracellular cytokine staining. In 10 VST products tested, we discovered 12 novel immunodominant epitopes. All products recognized an epitope at the C-terminus. On IFN-γ ELISpot, individual peptides eliciting activity demonstrated mean IFN-γ spot forming units per well (SFU)/1x10^5^ cells of 115.5 (range 24.5–247.5). VST products were polyfunctional, releasing IFN-γ and TNF-α in response to identified epitopes, which were primarily HLA Class II restricted. Peptides from Human Parainfluenza Virus-1 corresponding to the HPIV3 epitopes showed cross-reactivity for HPIV1 in 11 of 12 tested epitopes (mean cross reactivity index: 1.19). Characterization of HPIV3 epitopes may enable development of third-party VSTs to treat immune suppressed patients with HPIV infection.

## Introduction

Viral infections remain a major cause of morbidity and mortality in patients following hematopoietic stem cell transplantation (HSCT). Respiratory viruses, including human parainfluenza virus, can cause significant pulmonary disease and pneumonias in immunocompromised patients (1). Human parainfluenza virus 3 (HPIV3) is the most common serotype and is detected in 90% of infected patients. For patients with primary immunodeficiency disorders (PIDD) or patients post-transplant, HPIV3 can cause fatal pneumonia in up to 45% of infected patients (2, 3). Ribavirin and intravenous gamma globulin have been largely ineffective in lowering mortality, and currently, there are no effective anti-viral therapies nor approved vaccines for parainfluenza infections. Treatment therefore remains primarily symptom-based (4).

Susceptibility to viruses during the post-transplant period is due to compromised T cell immunity prior to full immune reconstitution. Adoptive T cell transfer has been highly successful in restoring viral-specific immunity in this patient population. For the past 20 years, virus-specific T-cells (VST) most commonly targeting cytomegalovirus (CMV) and Epstein Barr virus (EBV) have been used in numerous clinical trials (5, 6). Recent advances have led to the development of T cell products that target antigens from multiple viruses. (7–9) Identification of T-cells recognizing HPIV3 viral proteins led us to develop a Phase I study incorporating HPIV3 antigens in a 6 virus-specific T-cell product (NCT03180216). Pre-clinical data compared HPIV3-specific T-cell responses to four viral antigens (hemagglutinin-neuramidase, fusion, nucleocapsid, and matrix proteins) and identified that matrix, which is also highly conserved across sub-strains of HPIV3, elicited the most robust T-cell response (10). While most studies have used HSCT donors to generate VST, “off-the-shelf” T-cells from partially HLA-matched third party donors have also proven to be effective and reduce the cost and time delay prior to treatment (11, 12). Here we sought to identify immunodominant novel epitopes within HPIV3 Matrix protein to optimize the generation of potent HPIV-specific T-cells for adoptive therapy.

## Methods

### Donors

Peripheral blood mononuclear cells (PBMCs) from 10 healthy donors were obtained from Children’s National Hospital (Washington, DC) under informed consent approved by the Institutional Review Board in accordance with the Declaration of Helsinki. Eight donors had previously consented blood for virus-specific T cell manufacture for administration to patients enrolled on our IRB and FDA approved Phase I clinical trial evaluating a “first in human” multi-virus specific T cell product (NCT03180216). The remaining two donors were healthy volunteers who consented to our IRB approved blood procurement protocol (Pro00004033).

### Generation of VSTs

Evaluated T cell products included hexaviral-specific T-cells, manufactured from bone marrow transplant donors under protocol NCT03180216, and monovirus-specific HPIV3-VSTs produced from peripheral blood mononuclear cells (PBMCs) of healthy volunteers. In all cases, VSTs were produced using a rapid expansion protocol previously described (13). Briefly, PBMCs were pulsed with overlapping peptide pools encompassing viral antigens (200 ng/peptide/15 × 10^6^ PBMCs) for 30 min at 37°C. Peptide libraries for HPIV matrix and nucleoprotein were generated from the HPIV3 Wash/47885/57 reference sequence (JPT, Berlin, DE) (14). Hexaviral-specific T-cells were expanded against 12 antigens (CMV pp65/IE1, EBV LMP2/EBNA1, adenovirus Hexon/Penton, HHV6 U54/U90, BKV LgT/VP1, HPIV3 matrix/nucleoprotein), and monoviral HPIV3-specific T-cells were expanded against HPIV3 matrix and nucleoprotein only. After incubation, cells were resuspended with IL-4 (400 IU/ml; R&D Systems, Minneapolis, MN) and IL-7 (10 ng/ml; R&D Systems) in CTL media consisting of 45% RPMI (GE Healthcare, Logan, UT), 45% Click’s medium (Irvine Scientific, Santa Ana, CA), 10% fetal bovine serum, and supplemented with 2 mM GlutaMax (Gibco, Grand Island, NY). Cytokines were replenished on day 7. On day 10, cells were harvested and evaluated for antigen specificity and functionality.

### Anti-IFN-γ Enzyme-Linked Immunospot Assay

Antigen specificity of T-cells was measured by anti-IFNγ enzyme-linked immunospot ELISpot (Millipore, Burlington, MA). Ninety-six well filtration plates were coated overnight with 10 µg/ml anti-hu-IFN-mAb (capture mAB (1-DIK purified). T-cells (1x10^5^ cells/well) were stimulated with 15-mer peptide pools encompassing Matrix protein or individual 15-mers at 200 ng/peptide/well. For cross-reactivity testing, peptide libraries derived from human parainfluenza virus-1 (HPIV1) Matrix protein were generated using the Washington/1964 reference sequence. (REF) Staphylococcal enterotoxin-B (SEB) was used as a positive control (1 µg/ml), and actin was used as a negative control. Each condition was plated in duplicate or triplicate. After 24 h, the plates were washed, then incubated with the secondary biotin-conjugated anti-human-IFNγ−mAb (detector mAb (7-B6-1 biotin); Mabtech, Cincinnati, OH) at 1 µg/ml. After incubation with avidin:biotinylated HRP complex (Vectastain Elite ABC Kit (standard), PK6100; Vector Laboratories, Burlingame, CA), plates were developed with AEC substrate (A6926, Sigma-Aldrich, St Louis, MO). Frequency of T-cells specific for each peptide was expressed as IFN-γ spot-forming units (SFU), which were counted and evaluated using an automated plate reader system (Karl Zeiss; Zellnet Consulting, Fort Lee, NJ). Responses that were at least 20 SFU/1x10^5^ cells greater than background were considered positive.

HLA-restriction of epitope recognition was tested using blocking antibodies against HLA class I and II (Dako Agilent, Santa Clara, CA). To determine the HLA allelic restriction, VSTs were plated at 1 × 10^5^/well with partially HLA-matched phytohemagglutinin (PHA)-treated lymphoblasts (PHA-blasts) either alone or pulsed with peptide (10 μg/ml). Blocking of MHC class I and class II was performed by incubation of VST with polyclonal antibodies to human HLA-A/B/C or HLA-DR/DP/DQ for 1 h prior to plating for ELISpot assay as described above.

### Epitope Mapping

VSTs were tested for specificity to individual peptides within the Matrix protein by IFN- γ ELISpot. The Matrix 15-mer peptides were pooled using a matrix of 19 combinatorial peptide pools, **(****Supplemental Figure 1****)** These pools were designed so that each peptide is only represented in 2 pools. Cross-reactive pools were analyzed, and individual peptides were tested to confirm epitope specificity.

### Immunophenotyping

VSTs were stained with fluorophore-conjugated antibodies against CD3, CD4, CD8, CD14, CD16, CD19, CD45RO, CCR7, IFN-γ and TNF-α (Miltenyi Biotec, Bergisch Gladbach, Germany; BioLegend, San Diego, CA). All samples were acquired on a CytoFLEX cytometer (Beckman Coulter, Brea, CA). Data was analyzed with FlowJo X (FlowJo LLC, Ashland, OR) **(****Supplemental Figure 2****)**.

### Intracellular Cytokine Staining

A total of 0.5 to 1 × 10^6^ VSTs were plated in a 96-well U-bottom plate and stimulated with pooled pepmixes or individual peptides (200 ng/peptide/well) or actin (irrelevant peptide control) in the presence of Brefeldin A (Golgiplug; BD Biosciences, San Jose, CA) and CD28/CD49d (BD Biosciences) for 6 h. T-cells were fixed, stained with fluorophore-conjugated antibodies against CD4, CD8 and TCRαβ, permeabilized with Cytofix/Cytoperm solution (BD Biosciences), and stained with IFN−γ and TNF-α antibodies (Miltenyi Biotec).

### Data Analysis/Statistics

Results were evaluated using descriptive statistics (means, medians, standard deviations, ranges). Analysis was performed in GraphPad Prism (GraphPad software, La Jolla, CA).

## Results

### HPIV3-Specific T-Cells Comprise Polyclonal Populations With a CD4 Effector T Cell Predominance

**Figure 1:**
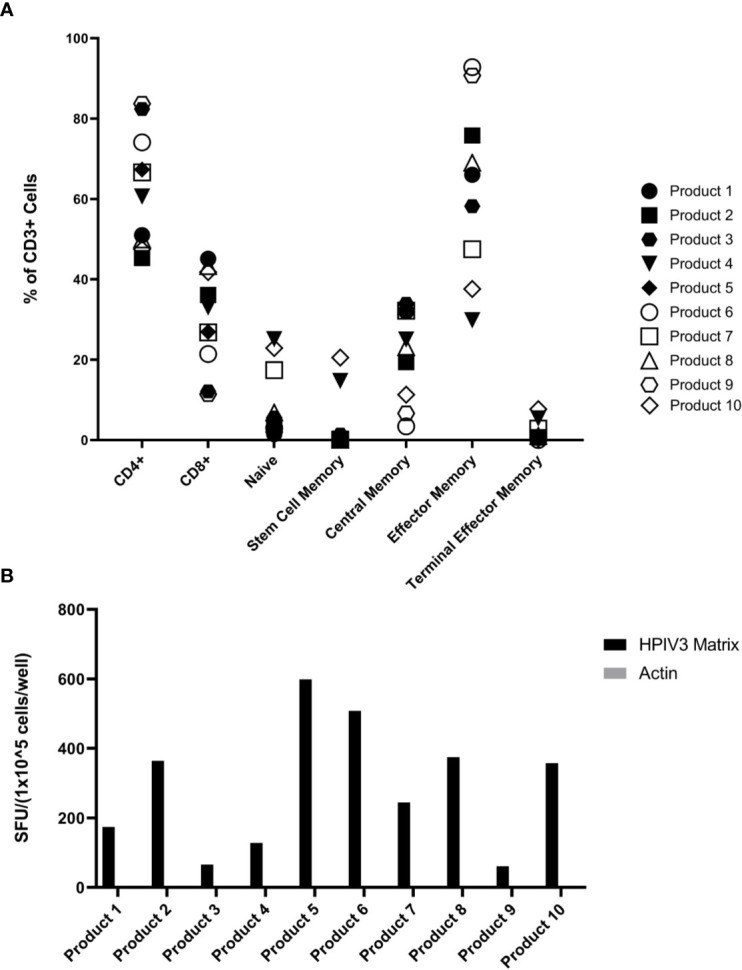
**(A)** Virus-specific T-cells (VST) Product Phenotyping. Phenotyping data of the hexaviral-specific T cell products specific for cytomegalovirus (CMV), Epstein Barr virus (EBV), AdV, HHV6, BKV, and HPIV3 (product #s 1-4, 6, 8-10) and monoviral-specific T cell products specific only for HPIV3 (product #s 5, 7) was obtained using flow cytometry. Phenotypes, including memory differentiation status panel, are reported as % of total CD3+ cells. Product #5 was unavailable for memory/differentiation phenotyping. Definitions of phenotypes are as follows: Naïve: CD45RA+CD45RO-CCR7+CD62L+CD95- Stem Cell Memory: CD45RA+CD45RO-CCR7+CD62L+CD95+ Central Memory: CD45RA-CD45RO+CCR7+CD62L+ Effector Memory: CD45RA-CD45RO+CCR7-CD62L- Terminal Effector Memory: CD45RA-CD45RO-CCR7-CD62L- **(B)** Overall HPIV3 Matrix specificity by product. All 10 viral-specific T cell products were stimulated with HPIV3 pepmix. Response was measured as spot forming units per well (SFU/1x10^5^ cells) by anti-IFN-γ ELISpot assay. Unstimulated T-cells (CTL only) and stimulation with actin pepmix (irrelevant antigen) were used as negative controls.

The hexaviral and monoviral HPIV3-expanded populations consisted of primarily T-cells (CD3+ median 95.75%, range 76.3%–98.9%). CD4+ T-cells comprised the majority of the expanded T-cells (median 63.6%; range 45.3%–83.7%) while CD8+ T-cells made up lesser proportions (median 30%; range 11.4%–45.1%) **(****Figure 1****)**. Differentiation status phenotyping of the CD3+ cells showed a predominance of effector memory cells (median 66%, range 29.8%–92.8%) **(****Supplemental Table 1****)**. All 10 products showed specificity for HPIV3 Matrix protein (Average IFN-γ spot forming units per well (SFU) 319.8/1x10^5^ cells) versus actin, an irrelevant antigen used as negative control. (SFU 0.5/1x10^5^ cells) **(****Figure 1B****)** *In silico* testing (Immune Epitope Database, IEDB.org) predicted that all 10 peptides would have MHC class II restricted responses primarily mediated through HLA-DRB1 alleles **(****Table 1****)**, and when stimulated with Matrix peptides, all products demonstrated a CD4^+^ response.

**Table 1:** HLA Class II alleles that identify peptide sequences of novel CD4^+^ T-cell epitopes.

Peptide #	Peptide sequence	Amino Acid Position	Product #	HLA-DRB1	HLA-DQB1	HLA-DPB1	IEDP MHC-II Binding Prediction
38	MLFDANKVALAPQCL	149−163	4	04:01, 07:01	03:02, 02:02	04:01, 17:01	DRB3*02:02
39	ANKVALAPQCLPLDR	153−167	4	04:01, 07:01	03:02, 02:02	04:01, 17:01	DRB1*01:01
50	SLPSTISINLQVHIK	197−211	7	11:01, **13:02**	03:01, 06:04	04:01, 14:01	DRB1*13:02
59	LNFMVHLGLIKRKVG	233−247	3	07:01, **11:01**	02:02, 03:01	10:01, 23:01	DRB1*11:01
			5	04:04, **11:01**	03:01, 03:02	04:01, 04:01	
60	VHLGLIKRKVGRMYS	237−251	5	04:04, **11:01**	03:01, 03:02	04:01, 04:01	DRB1*11:01
			7	**11:01**, 13:02	03:01, 06:04	04:01, 14:01	
76	PLMDLNPHLNLVIWA	301−315	3	07:01, 11:01	02:02, 03:01	10:01, 23:01	DRB1*12:01
77	LNPHLNLVIWASSVE	305−319	1	03:01, 08:02	02:01, 04:01	01:01, 04:02	DRB1*04:05
			2	07:FKP, 11:04	02:ANKDG, 03:APAKC	01:01, 04:01	
78	LNLVIWASSVEITRV	309−323	2	07:FKP, 11:04	02:ANKDG, 03:APAKC	01:01, 04:01	DRB1*13:02
82	AIFQPSLPGEFRYYP	325−339	1	03:01, 08:02	02:01, 04:01	01:01, 04:02	DRB1*09:01
			4	04:01, 07:01	03:02, 02:02	04:01, 17:01	
			5	04:04, 11:01	03:01, 03:02	04:01, 04:01	
			6	15:01, 15:01	06:02, 06:02	04:01, –	
			7	11:01, 13:02	03:01, 06:04	04:01, 14:01	
			8	07:FKP, 07:FKP	03:AJRDT, 03:AJDZN	04:01, –	
			10	04:02, 15:01	03:02, 06:02	02:01, 04:01	
83	PSLPGEFRYYPNIIA	329−343	1	03:01, 08:02	02:01, 04:01	01:01, 04:02	DRB1*15:01
			2	07:FKP, 11:04	02:ANKDG, 03:APAKC	01:01, 04:01	
			3	07:01, 11:01	02:02, 03:01	10:01, 23:01	
			4	04:01, 07:01	03:02, 02:02	04:01, 17:01	
			5	04:04, 11:01	03:01, 03:02	04:01, 04:01	
			6	**15:01, 15:01**	06:02, 06:02	04:01, –	
			7	11:01, 13:02	03:01, 06:04	04:01, 14:01	
			8	07:FKP, 07:FKP	03:AJRDT, 03:AJDZN	04:01, –	
			10	04:02, **15:01**	03:02, 06:02	02:01, 04:01	
84	GEFRYYPNIIAKGVG	333−347	1	03:01, 08:02	02:01, 04:01	01:01, 04:02	DRB1*15:01
			2	07:FKP, 11:04	02:ANKDG, 03:APAKC	01:01, 04:01	
			3	07:01, 11:01	02:02, 03:01	10:01, 23:01	
			4	04:01, 07:01	03:02, 02:02	04:01, 17:01	
			5	04:04, 11:01	03:01, 03:02	04:01, 04:01	
			6	**15:01, 15:01**	06:02, 06:02	04:01, –	
			7	11:01, 13:02	03:01, 06:04	04:01, 14:01	
			8	07:FKP, 07:FKP	03:AJRDT, 03:AJDZN	04:01, –	
			9	03:02, **15:03**	02:03, 06:–	–, –	
			10	04:02, **15:01**	03:02, 06:02	02:01, 04:01	
85	YYPNIIAKGVGKIKQ	337−351	2	07:FKP, 11:04	02:ANKDG, 03:APAKC	01:01, 04:01	DRB1*08:02
			4	04:01, 07:01	03:02, 02:02	04:01, 17:01	

HLA alleles that match the IEDP MHC-II Binding Prediction are listed in bold.

### HPIV3-Specific CD4+ T-Cells Respond to Multiple Class II-Restricted Epitopes Within Matrix Protein

**Figure 2:**
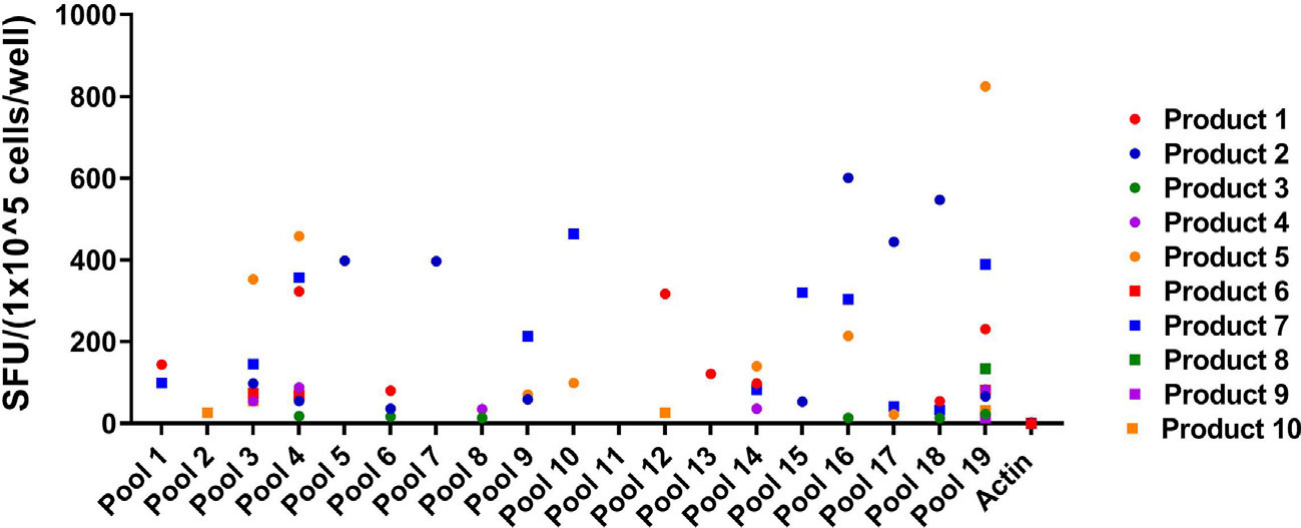
Peptide Pool specificity by product. Virus-specific T-cells (VSTs) were stimulated with combinatorial HPIV3 peptide pools. Response was measured as spots per well (SFU/1x10^5^ cells) by anti-IFN-γ ELISpot assay. Unstimulated T-cells (CTL only) and stimulation with actin (irrelevant antigen) were used as negative controls.

Eighteen of the 19 pools were recognized by at least one of the VST products (SFU > 10/1x10^5^ cells), and three of the pools were recognized by five or more VST products **(****Figure 2****)**. Twelve novel epitopes were recognized among the 10 different T cell products- 38, 39, 50, 59, 60, 76, 77, 78, 82, 83, 84, 85 **(****Table 1****)**. All 10 products recognized peptide 84 (GEFRYYPNIIAKGVG; median SFU 151.9/1x10^5^ cells (range 24–564); mean SFU 184.9/1x10^5^ cells); nine products recognized peptide 83 (PSLPGEFRYYPNIIA; median SFU 79.8/1x10^5^ cells (range 36–484); mean SFU 141.1/1x10^5^ cells); and seven products recognized peptide 82 (AIFQPSLPGEFRYYP; median SFU 43/1x10^5^ cells (range 22–443); mean SFU 107.1/1x10^5^ cells). The remaining nine epitopes were recognized by one to three products **(****Table 2****)**. None of the products recognized actin [Median SFU 0.5/1×10^5^ cells (range 0–1)].

**Table 2:** Hierarchy of immunodominance of HPIV3 Matrix epitopes.

Peptide #	Amino acid sequence	Epitope	Responders (n=10)	Mean SFU ± Standard Dev	Median SFU (Range)
84	333-347	GEFRYYPNIIAKGVG	10	184.9 ± 160	151.9 (24−564)
83	329-343	PSLPGEFRYYPNIIA	9	141.1 ± 136	79.8 (36−484)
82	325-339	AIFQPSLPGEFRYYP	7	107.1 ± 143	43 (22−443)
59	233-247	LNFMVHLGLIKRKVG	2	84.3 ± 97	16 (14−152.5)
85	337-351	YYPNIIAKGVGKIKQ	2	115.3 ± 75	115.3 (62−168.5)
60	237-251	VHLGLIKRKVGRMYS	2	145.5 ± 6	145.5 (141.5−149.5)
76	301-315	PLMDLNPHLNLVIWA	2	24.5 ± 3.5	24.5 (22−27)
78	309-323	LNLVIWASSVEITRV	1	247.5	247.5
38	149-163	MLFDANKVALAPQCL	1	117.5	117.5
77	305-319	LNPHLNLVIWASSVE	1	87.5	87.5
39	153-167	ANKVALAPQCLPLDR	1	77	77
50	197-211	SLPSTISINLQVHIK	1	54	54

### HPIV3 Epitopes Demonstrate Cross-Reactivity With HPIV1

**Figure 3:**
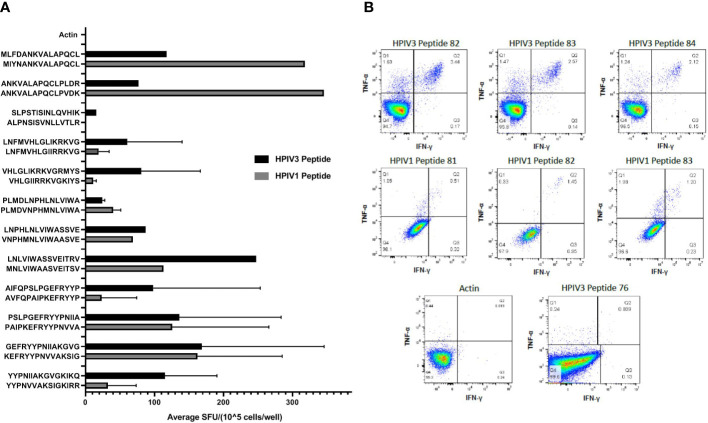
**(A)** Cross reactivity between HPIV3 and HPIV1. Virus-specific T-cells (VSTs) were stimulated with individual HPIV3 peptides and corresponding HPIV1 peptides. Response was measured as spots per well (SFU/1x10^5^ cells) by anti-IFN-γ ELISpot assay, and mean responses were calculated among responding products. Black bars represent HPIV3 peptides and gray bars represent HPIV1 peptides. **(B)** CD4+ T Cell Responses against HPIV3 and HPIV1 peptides. Intracellular flow cytometry was performed on VSTs following stimulation with Actin, SEB, HPIV3 or HPIV1 peptides and anti-CD28/CD49. After 2 h stimulation with peptides, Brefeldin A was added for an additional 4 h following which cells were labeled with dead cell exclusion dye, Fc Receptor block and antibodies against CD3, CD4, CD8, TNF-α, IFN-γ, and CD95. Following fixation and permeabilization cells were labeled intracellularly with antibodies targeting TNF-α, IFN-γ, Percentage of CD4+ and CD8+ T-cells producing both IFNγ+ and/or TNFα+ were measured.

Given the homology between HPIV3 and related Respirovirus Human parainfluenza virus 1 (3), we sought to determine if immunodominant epitopes identified within HPIV3 Matrix protein were cross reactive with corresponding Human Parainfluenza Virus 1 epitopes. We initially tested overall specificity of HPIV3 specific T cell products for HPIV1 Matrix pepmix compared to HPIV3 Matrix pepmix using anti-IFN-γ ELISpot **(****Supplemental Figure 3****)**. The mean response from all 10 products elicited by HPIV3 pepmix was 319.8 SFU/1x10^5^ cells, while the mean response elicited by HPIV1 pepmix was 138.9 SFU/1 x10^5^ cells. Median actin, used as negative control, was 0.5 SFC/1×10^5^ cells (range 0–1). This resulted in a cross-reactivity index (HPIV1 response/HPIV3 response) of 0.4. We evaluated the cross-reactivity of HPIV3 specific T-cells against HPIV1 peptides using anti-IFN-γ ELISpot. Cross-reactivity indices (HPIV1 mean SFU/HPIV3 mean SFU) ranged from 0.03 to 4.5 **(****Table 3****)**. There was a high level of cross-reactivity between HPIV3 Peptides 83-84 and HPIV1 Peptides 82-83, with a cross-reactivity index of 1 and 1.07, respectively. HPIV1 Peptides 37 and 38 elicited a stronger response from HPIV3 specific T-cells than the corresponding HPIV3 epitopes, with cross-reactivity indices of 2.71 and 4.5, respectively. Of the 12 identified epitopes, 7 to 13 out of 15 amino acid sequences were conserved between strains **(****Figure 3A****)**.

**Table 3:** Cross reactivity indices of HPIV3 Matrix epitopes and homologous HPIV1 Matrix epitopes.

HPIV3 matrix epitope	Amino acid sequence	Corresponding HPIV1 matrix epitope	Amino acid sequence	Shared amino acids	Cross reactivity index
Peptide 38	MLFDANKVALAPQCL	Peptide 37	M**IYN**ANKVALAPQCL	12	2.71
Peptide 39	ANKVALAPQCLPLDR	Peptide 38	ANKVALAPQCLP**V**D**K**	13	4.5
Peptide 50	SLPSTISINLQVHIK	Peptide 49	**A**LP**NS**IS**V**NL**L**V**TLR**	7	0.03
Peptide 59	LNFMVHLGLIKRKVG	Peptide 58	LNFMVHLG**I**I**R**RKVG	13	0.3
Peptide 60	VHLGLIKRKVGRMYS	Peptide 59	VHLG**I**I**R**RK**V**G**KI**YS	10	0.28
Peptide 76	PLMDLNPHLNLVIWA	Peptide 75	PLMD**V**NPH**M**NLVIWA	13	1.64
Peptide 77	LNPHLNLVIWASSVE	Peptide 76	**V**NPH**M**NLVIWA**A**SVE	12	0.78
Peptide 78	LNLVIWASSVEITRV	Peptide 77	**M**NLVIWA**A**SVEIT**S**V	12	0.46
Peptide 82	AIFQPSLPGEFRYYP	Peptide 81	A**V**FQP**AI**P**K**EFRYYP	11	0.27
Peptide 83	PSLPGEFRYYPNIIA	Peptide 82	P**AI**P**K**EFRYYPN**VV**A	10	1
Peptide 84	GEFRYYPNIIAKGVG	Peptide 83	**K**EFRYYPN**VV**AK**SI**G	10	1.07
Peptide 85	YYPNIIAKGVGKIKQ	Peptide 84	YYPN**VV**AK**SI**GKI**RR**	9	0.28

Cross-reactivity index = variant response/original response. Variation sequences are indicated in bold.

Intracellular cytokine staining flow cytometry showed a predominant CD4^+^ response to Matrix peptides 38^–^39, 50, 59–60, 77–78, and 82–85 **(****Figure 3B****)**. Responding CD4+ cells demonstrated polyfunctional cytokine production, with a mean of 0.77% of cells producing IFN-γ and TNF-α (range 0.03%–3.44%) compared to 0.09% of CD8+ cells (range 0%–0.39%). Peptide 76 elicited a weak CD8+ response (mean 0.37%± 0.015%) **(****Supplemental Table 2****)**. HPIV1 Matrix peptides 81, 82, and 83 showed a mixed response, with both CD4+ cells (0.82% ± 0.3%) and CD8+ cells (1.34% ± 0.8%) producing both IFN- γ and TNF-α (**Figure 3B**).

### HPIV3 Epitopes Elicit an MHC Class II-Restricted Response

**Figure 4:**
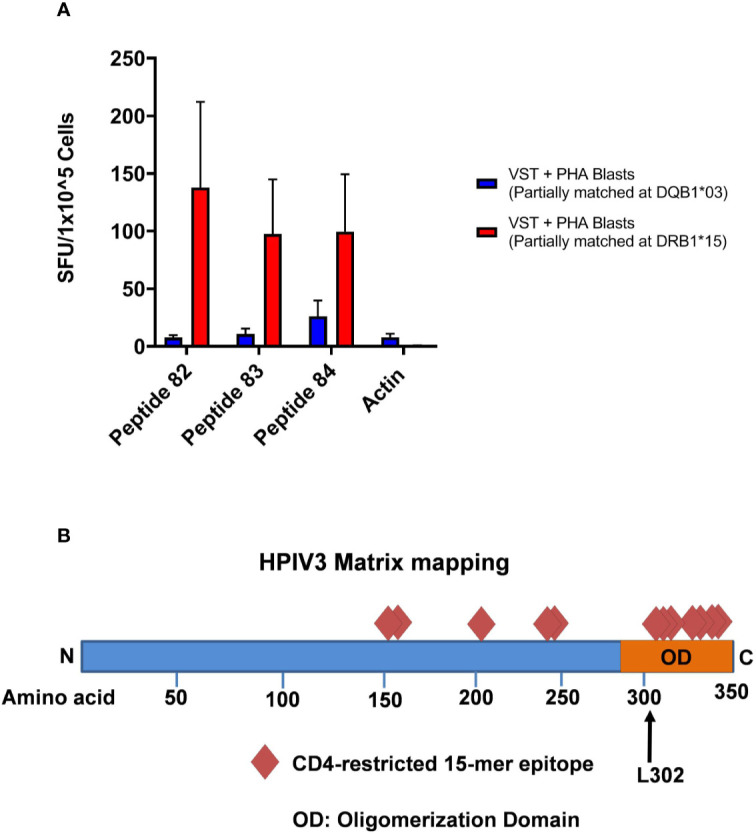
**(A)** HLA Restriction Mapping. Two phytohemagglutinin (PHA) blast lines (each partially matched to a Virus-specific T-cell (VST) product at HLA-DR*15:01 and HLA-DQ*03:01, respectively) were pulsed with HPIV3 peptides 82, 83, and 84 for 60 min, triple washed, and then presented to VSTs. T cell activity was detected using anti-IFN-γ ELISpot. **(B)** HPIV3 Matrix diagram with epitopes and C-terminal domain. HPIV3 Matrix protein contains 353 amino acids. Diamonds represent the novel epitopes identified during mapping. The oligomerization domain at the C-terminus contains 7 out of 12 epitopes, including the three most immunodominant peptides, 82–84. The black arrow denotes the L302 residue, which is a target of ubiquitination during virion production.

HLA blocking antibodies were used in the presence of HPIV3 specific T-cells and peptides 82, 83, and 84. IFN- γ production by ELISpot was decreased by a mean of 56.5% (range 36.3%–80.4%) with anti-HLA-DR antibodies. Restriction analysis of peptides 82, 83, and 84 using anti-IFN-γ ELISpot showed that VSTs responded to peptide-pulsed PHA blast targets matched at HLA-DRB1*15:01, with mean SFU/1x10^5^ cells of 137.7, 97.5, and 99.7 (when presenting 82, 83, and 84, respectively) compared to PHA blasts and peptides alone (mean SFU/1x10^5^ cells of <1) **(****Figure 4A****)**. This was in line with *in silico* predictions of MHC binding of the peptides. In comparison, restriction analysis was performed using VSTs and PHA blast targets partially matched at HLA-DRQ1*03:01. VSTs did not respond to peptides presented by these PHA blasts, with mean SFU/1x10^5^ cells decreased by 93%–95%.

## Discussion

Human parainfluenza virus infections in immunocompromised patients remain a significant cause of morbidity and mortality and lack specific anti-viral treatment. Virus-specific T-cells have proven effective in treatment of several viral infections occurring post-stem cell transplantation and our preclinical studies have shown that HPIV3 specific T-cells can be produced from healthy donors and successfully target HPIV3 epitopes (10). Further defining VSTs targeting HPIV3 will expand their applicability as third party products. Because transplant donor-derived VST production is limited when the donor is unrelated or the stem cell source is cord blood, and it takes many days to generate the product, “off-the-shelf” VST could be used as immediately available products to treat a number of viruses (11, 15, 16). However, this approach has not yet been applied to HPIV3 infections. Hence, identification of immunodominant HPIV3 epitopes is essential for selection of partially-HLA matched VSTs, and development of third-party HPIV3 VST therapy.

In this study, we identified 12 new epitopes within the HPIV3 Matrix protein, which were recognized by multiple donors. We discovered a highly dominant epitope within the group, GEFRYYPNIIAKGVG, which elicited anti-viral activity from all 10 products. Overlapping 15mers AIFQPSLPGEFRYYP and PSLPGEFRYYPNIIA also produced responses to 7 and 9 out of 10 products, respectively. This cluster of epitopes resides at the C-terminus, containing a leucine residue critical for regulating virion release **(****Figure 4B****)** (17). Although there has been limited success to date with parainfluenza vaccinations, the identification of this immunogenic epitope cluster may also have significance for future vaccine development (18, 19).

We determined that the identified epitopes are HLA class II restricted, similar to the predominance of CD4-restricted responses against the adenovirus Hexon protein and BK virus large T antigen (11, 12, 20, 21). These findings reiterate the importance of CD4^+^ T cells in their role of respiratory virus clearance. Epitopes induced polyfunctional responses based on TNF-α and IFN-γ production. *In silico* predictions suggested that the most immunodominant peptide, GEFRYYPNIIAKGVG, would elicit a T cell response restricted through HLA-DRB1*15:01. Only 2 out of 10 products were matched at that allele, but restriction analysis showed that the VSTs responded to PHA blast targets matched at HLA-DRB1*15:01. We also showed that IFN-γ production was decreased by 32%–80% in the presence of HLA-DR blocking antibodies. Similar findings were noted in CD4+ responses against MHC Class II-restricted adenovirus Hexon epitopes, where the T-cell response of multiple T cell products with differing HLA-alleles was blocked by an HLA-DR antibody (22). Our findings suggest that while there is some degree of HLA-DR restriction, T cell activity against GEFRYYPNIIAKGVG is likely not limited to a single Class II allele. Although our products were manufactured from a diverse group of donors, a limitation to this study is that we cannot evaluate for all possible HLA restricted responses. However, we and others have shown that even knowing a relatively restricted number of class I and class II restricted virus-specific responses provides important clinical information when evaluating virus-specific T cell products in a third party “off the shelf” setting (16, 23).

Previous studies have shown that cytotoxic T-cells stimulated by HPIV1-infected cells also recognize HPIV3 proteins (24). Additional studies showed that while antibody cross-reactivity is minimal within the paramyxovirus family, T cell cross-reactivity is common between HPIV3 and other related viruses such as measles, mumps, and RSV (25). Since HPIV3 and HPIV1 both occur in an immunocompromised population, we tested peptide sequences from HPIV1 corresponding to the amino acid sequences from the identified HPIV3 epitopes, and confirmed cross-reactivity in 10 of 12 epitopes. Two of the HPIV1 peptides elicited a more robust T cell response than their homologous HPIV3 epitopes in one subject, which suggests that the elicited T-cell response from the donor may have been derived from a recent immunologic response to HPIV1 rather than to HPIV3. The highest degree of cross-reactivity was between HPIV3 epitopes 83 and 84 and the corresponding HPIV1 epitopes (82 and 83, respectively), which were the epitopes recognized most consistently by the T cell products. Though the clinical impact of T-cells targeting these identified epitopes is not yet known, their immunodominance and cross-reactivity with HPIV1 argue that T-cell response to these epitopes serves an important role in the adaptive immune response to human parainfluenza viruses.

In conclusion, we have characterized multiple novel immunodominant epitopes within HPIV3 Matrix protein. These HPIV3 epitopes show a high degree of cross-reactivity with corresponding HPIV1 epitopes. Our study paves the way for developing third party HPIV3 and HPIV1- VST products to rapidly treat immunocompromised individuals infected with these lethal respiratory viruses.

## Data Availability Statement

All datasets presented in this study are included in the article/**Supplementary Material**.

## Ethics Statement

The studies involving human participants were reviewed and approved by: the Children’s National Hospital IRB (Protocol IRB # Pro00008637). Written informed consent to participate in this study was provided by the participant, or participants’ legal guardian.

## Author Contributions

KH, SH, VK, MJ-W, GS, HL, CL, and AD contributed by performing lab experiments including cell culture, intracellular cytokine staining flow cytometry and ELISpot, as well as assisted with data analysis. MK and CB contributed by providing mentorship, laboratory leadership, experiment design, and data analysis. All authors contributed to the article and approved the submitted version.

## Funding

This work was supported by grants from the National Institutes of Health (K23-HL136783-01 to MK), the Jeffrey Modell Foundation, and the Board of Visitors of the Children’s National Health System.

## Conflict of Interest

CB is on the scientific advisory board for Cellectis and has stock options in NexImmune and Torque Therapeutics and has stock or ownership in Mana Therapeutics. MK is on a scientific advisory board for Gilead Sciences. KH, CB, and MK have filed a patent application based on the findings in this paper.

The remaining authors declare that the research was conducted in the absence of any commercial or financial relationships that could be construed as a potential conflict of interest.

## Supplementary Material

Supplementary Figure 1 Combinatorial peptide pools HPIV3 Matrix peptides were pooled according to this matrix. Cross-reactive pools were analyzed and individual peptides were tested to confirm epitope specificity, as each peptide is uniquely represented in two specific pools.

Supplementary Figure 2 Gating Strategy. Compensation was performed using antibody capture beads (all antibodies) and cells (viability dye). Cells were acquired on a Beckman Coulter CytoFlex S using CytExpert version 2.2.0.97 software. Data was analyzed on Flow Jo version 10.5. Cells are first gated as singlets, then live cells. CD8+/CD4+/CD3-/CD3+CD4-CD8- cell were interrogated identically using bivariate plots assessing IFN-γ and TNF-α. Where > 8% of events fell on an axis bi-exponential scaling was used to visualize all cells on the plot.

Supplementary Figure 3 Overall HPIV3 and HPIV1 Matrix specificity by product All 10 viral-specific T cell products were stimulated with both HPIV3 pepmix and HPIV1 pepmix. Response was measured as spots per well (SFU/1x10^5^ cells) by anti-IFN-γ ELISpot assay. Unstimulated T-cells (CTL only) and stimulation with actin pepmix (irrelevant peptide) were used as negative controls.

Supplementary Table 1 VST Product Phenotyping showing percentages of various populations within expanded T cells.

Supplementary Table 2 Percentages of T cells producing both IFN-γ and TNF-α
