# Supplementary figures and images for: T-Cell Therapeutics Targeting Human Parainfluenza Virus 3 Are Broadly Epitope Specific and Are Cross Reactive With Human Parainfluenza Virus 1
Source: Front Immunol. 2020 Oct 5;11:575977. doi: 10.3389/fimmu.2020.575977 (PMC7573487; doi:10.3389/fimmu.2020.575977)

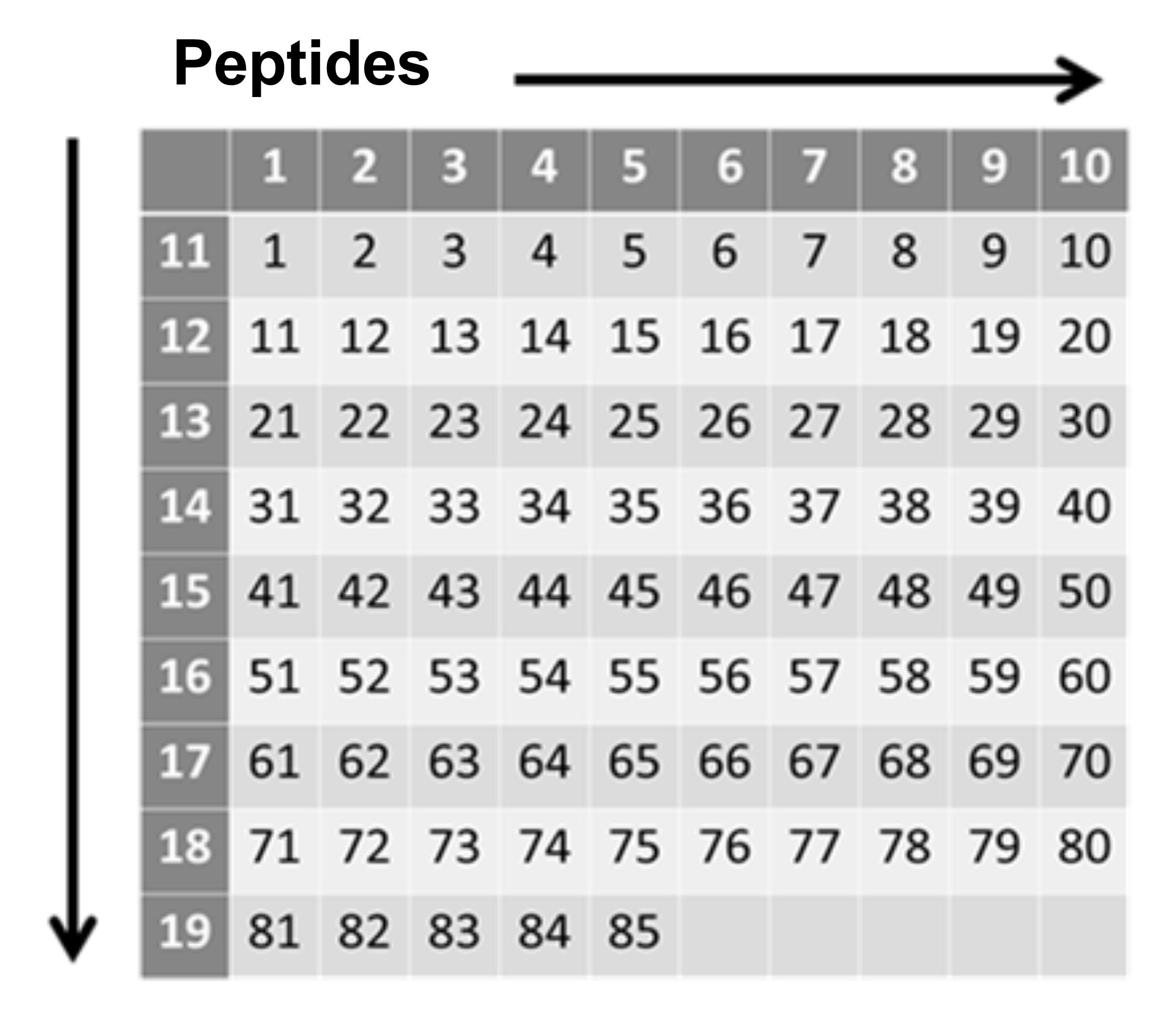

Supplement: Supplementary Figure 1 — Combinatorial peptide pools HPIV3 Matrix peptides were pooled according to this matrix. Cross-reactive pools were analyzed and individual peptides were tested to confirm epitope specificity, as each peptide is uniquely represented in two specific pools. [file Image_1.jpeg]

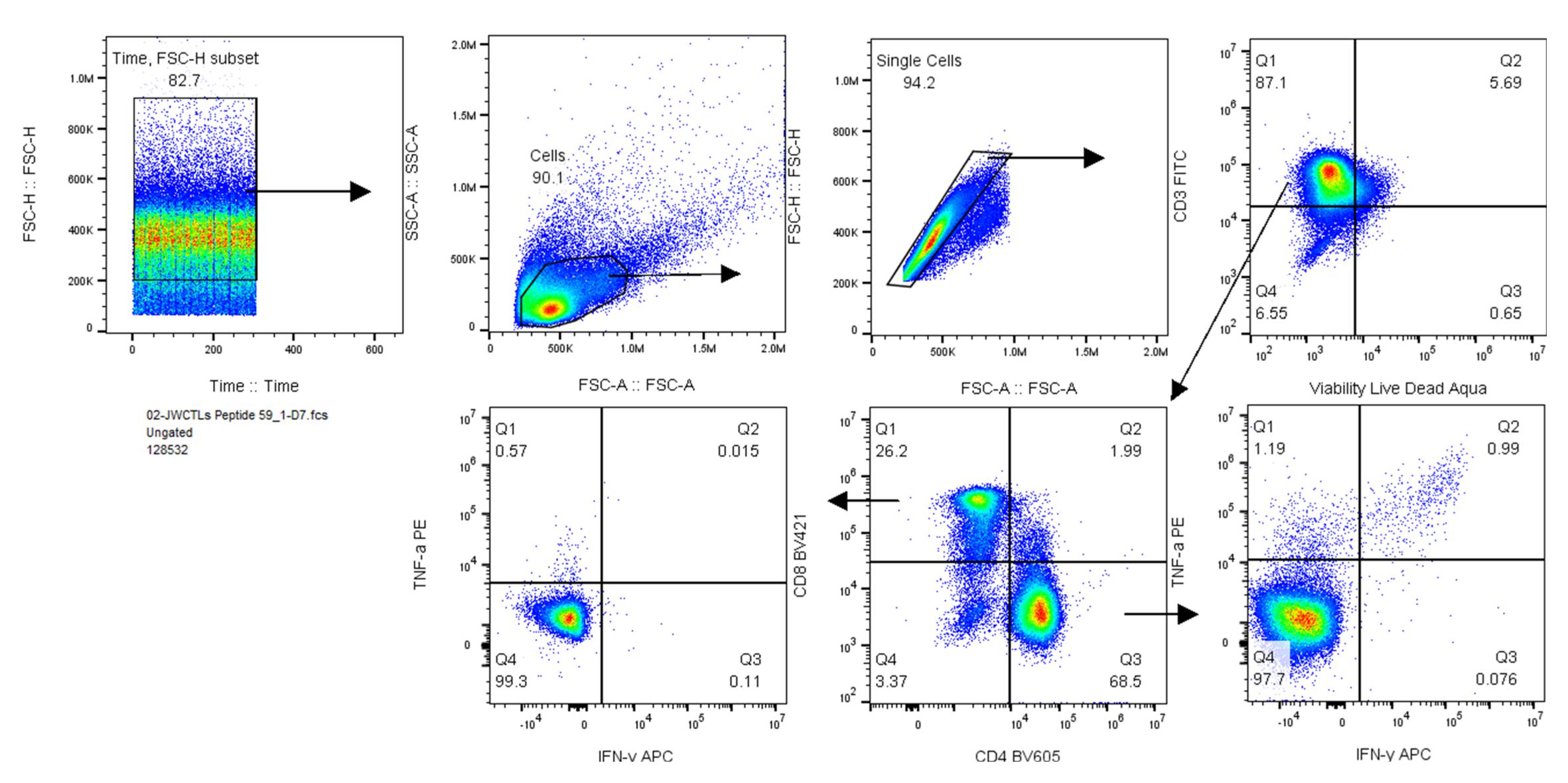

Supplement: Supplementary Figure 2 — Gating Strategy. Compensation was performed using antibody capture beads (all antibodies) and cells (viability dye). Cells were acquired on a Beckman Coulter CytoFlex S using CytExpert version 2.2.0.97 software. Data was analyzed on Flow Jo version 10.5. Cells are first gated as singlets, then live cells. CD8+/CD4+/CD3-/CD3+CD4-CD8- cell were interrogated identically using bivariate plots assessing IFN-γ and TNF-α. Where > 8% of events fell on an axis bi-exponential scaling was used to visualize all cells on the plot. [file Image_2.jpeg]

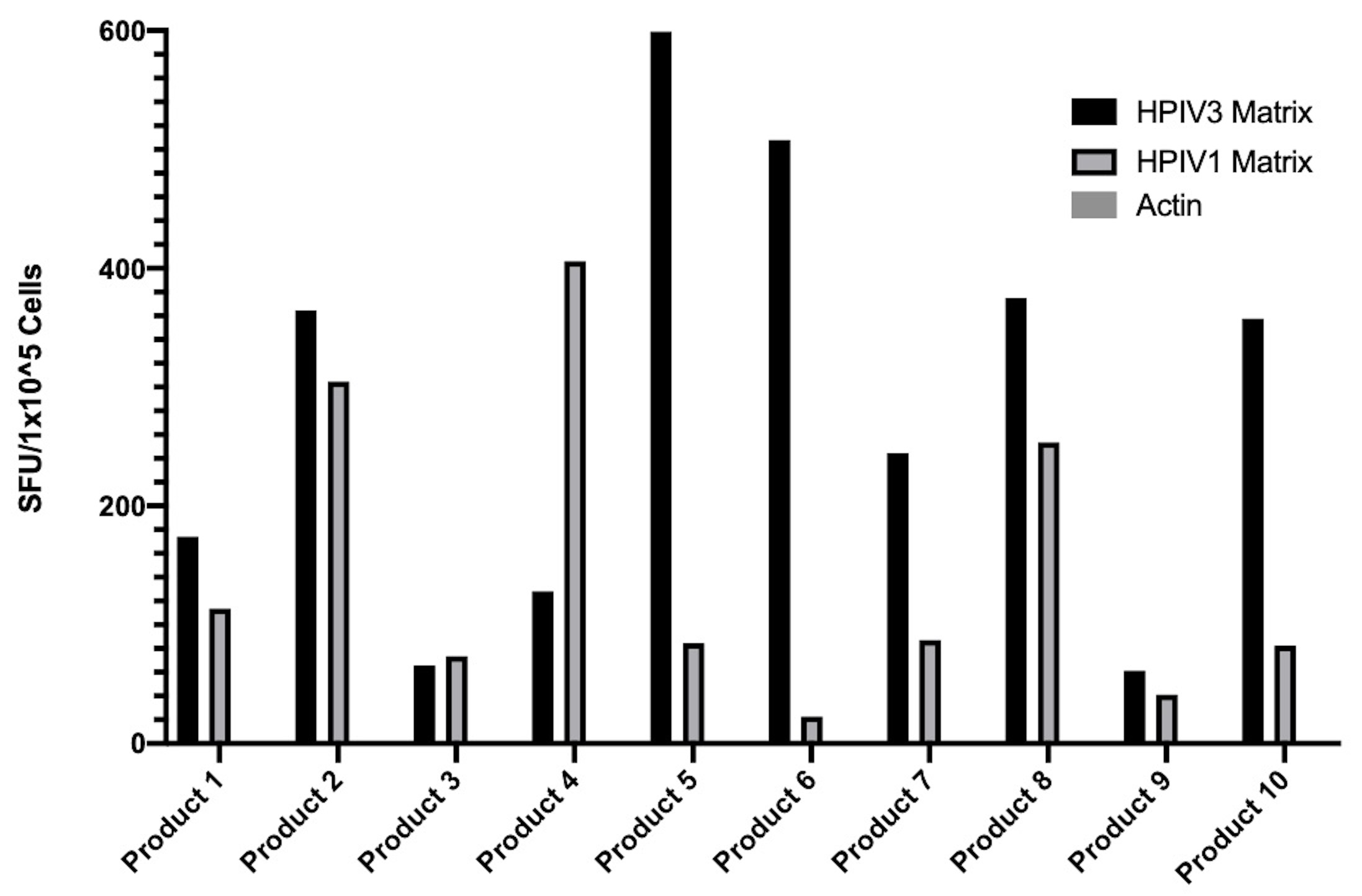

Supplement: Supplementary Figure 3 — Overall HPIV3 and HPIV1 Matrix specificity by product All 10 viral-specific T cell products were stimulated with both HPIV3 pepmix and HPIV1 pepmix. Response was measured as spots per well (SFU/1x105 cells) by anti-IFN-γ ELISpot assay. Unstimulated T-cells (CTL only) and stimulation with actin pepmix (irrelevant peptide) were used as negative controls. [file Image_3.jpeg]
