# Supplementary material for: T-Cell Therapeutics Targeting Human Parainfluenza Virus 3 Are Broadly Epitope Specific and Are Cross Reactive With Human Parainfluenza Virus 1
Source: Front Immunol. 2020 Oct 5;11:575977. doi: 10.3389/fimmu.2020.575977 (PMC7573487; doi:10.3389/fimmu.2020.575977)
Supplement: Supplementary Table 1 — VST Product Phenotyping showing percentages of various populations within expanded T cells. [file Table_1.docx]

**Supplemental Table 1:
VST Product Phenotyping showing percentages of various populations within expanded T cells**

| **Product #** | **1** | **2** | **3** | **4** | **5** | **6** | **7** | **8** | **9** | **10** |
| --- | --- | --- | --- | --- | --- | --- | --- | --- | --- | --- |
| **% CD3+** | **96.7** | **83.8** | **94.8** | **96.7** | **76.28** | **96.7** | **87.25** | **89.8** | **96.7** | **98.9** |
| % CD3+/CD4+ | 51 | 45.3 | 82.4 | 60.6 | 67.3 | 74.1 | 66.6 | 49.9 | 83.7 | 47.5 |
| %  CD3+/CD8+ | 45.1 | 36.1 | 12.1 | 33 | 26.9 | 21.4 | 26.8 | 43.3 | 11.4 | 41.8 |
| %  CD45RA+CD45RO-CCR7+CD62L+CD95- | 1.48 | 3.75 | 5.59 | 25.1 | - | 3 | 17.4 | 6.82 | 1.98 | 22.9 |
| %  CD45RA+CD45RO-CCR7+CD62L+CD95+ | 0.08 | 0.37 | 1.34 | 14.7 | - | 0.2 | 0.11 | 0.05 | 0.15 | 20.5 |
| %  CD45RA-CD45RO+  CCR7+CD62L+ | 32 | 19.4 | 33.9 | 25 | - | 3.4 | 32.2 | 23.1 | 6.65 | 11.3 |
| %  CD45RA-CD45RO+  CCR7-CD62L- | 66 | 75.8 | 58.2 | 29.8 | - | 92.8 | 47.5 | 69 | 90.7 | 37.6 |
| %  CD45RA-CD45RO-  CCR7-CD62L- | 0.49 | 0.72 | 1 | 5.37 | - | 0 | 2.8 | 1.04 | 0.54 | 7.7 |

| HPIV3 Matrix Epitope | Mean % CD4+ Response  (IFNγ+/TNFα+) | Mean % CD8+ Response  (IFNγ+/TNFα+) |
| --- | --- | --- |
| Peptide 38 | 0.62 | 0.07 |
| Peptide 39 | 0.45 | 0.05 |
| Peptide 50 | 1.44 | 0 |
| Peptide 59 | 0.48 | 0.03 |
| Peptide 60 | 0.25 | 0.19 |
| Peptide 76 | 0.043 | 0.38 |
| Peptide 77 | 0.28 | 0.045 |
| Peptide 78 | 0.79 | 0.15 |
| Peptide 82 | 1.74 | 0.01 |
| Peptide 83 | 1.58 | 0.01 |
| Peptide 84 | 0.88 | 0.13 |
| Peptide 85 | 0.65 | 0.04 |

**Supplemental Table 2: Percentages of T cells producing both IFN-γ and TNF-α**

**Supplemental Figure Legend**

**Supplemental Figure 1: Combinatorial peptide pools**HPIV3 Matrix peptides were pooled according to this matrix. Cross-reactive pools were analyzed and individual peptides were tested to confirm epitope specificity, as each peptide is uniquely represented in two specific pools.

**Supplemental Figure 2: Gating Strategy**.

Compensation was performed using antibody capture beads (all antibodies) and cells (viability dye). Cells were acquired on a Beckman Coulter CytoFlex S using CytExpert version 2.2.0.97 software. Data were analyzed on Flow Jo version 10.5. Cells are first gated as singlets, then live cells. CD8+/CD4+/CD3-/CD3+CD4-CD8- cell were interrogated identically using bivariate plots assessing IFN-γ and TNF-α. Where > 8% of events fell on an axis bi-exponential scaling was used to visualize all cells on the plot.

**Supplemental Figure 3: Overall Matrix specificity by product**
Hexaviral specific T cell products specific for CMV, EBV, AdV, HHV6, BKV, and HPIV3 (product #s 1-4, 6, 8-10) and monoviral specific T cell products specific only for HPIV3 (product #s 5, 7) were stimulated HPIV3 and HPIV1 pepmixes. Response was measured as spots per well (SPW/1x10^5 cells) on IFN-γ ELISpot assay. Unstimulated T cells (CTL only) and stimulation with actin pepmix were used as negative controls.
